# Assessing the online search behavior for COVID-19 outbreak: Evidence from Iran

**DOI:** 10.1371/journal.pone.0267818

**Published:** 2022-07-26

**Authors:** Mahnaz Samadbeik, Ali Garavand, Nasim Aslani, Farzad Ebrahimzadeh, Farhad Fatehi

**Affiliations:** 1 Social Determinants of Health Research Center, Lorestan University of Medical Sciences, Khorramabad, Iran; 2 Department of Biostatistics and Epidemiology, School of Health and Nutrition, Nutritional Health Research Center, Lorestan University of Medical Sciences, Khorramabad, Iran; 3 School of Psychological Sciences, Monash University, Melbourne, Australia; 4 Centre for Health Services Research, The University of Queensland, Brisbane, Australia; Istanbul University Istanbul Faculty of Medicine: Istanbul Universitesi Istanbul Tip Fakultesi, TURKEY

## Abstract

**Introduction:**

Google Trends (GT) is an important free tool for online search behavior analysis, which provides access to Internet search patterns in Google. In recent decades, this database has been used for predicting the outbreak of epidemics and pandemics in different regions of the world. The present study aimed to evaluate Iranian users’ COVID-19-related online search behavior.

**Methods:**

This longitudinal study was conducted in 2021. The data of Iranian users’ COVID-19-related online search behavior (trend) were collected from the GT website, and the epidemiological data of the COVID-19 outbreak in Iran from 16 February 2020 to 2 January 2021 were sourced from the Iranian ministry of health and medical education, as well as the World Health Organization. The data were analyzed in SPSS using descriptive and inferential statistics.

**Results:**

All the COVID-19-related search terms in Iran gained their highest popularity value (relative search volume = 100) in the first 8 weeks of the pandemic, and then this value assumed a decreasing trend over time. Based on factor analysis, relative search volume (RSV) of factor 1 terms (related to corona [in Persian] and corona) have a low significance relationship with COVID-19 epidemiological data in one-, two-, and three-week time lags. Although, RSV of factor 2 terms (related to COVID [in Persian], COVID-19, and coronavirus) correlated with the total weekly number of COVID-19 cases in mentioned time lags.

**Conclusion:**

COVID-19-related search terms were popular among Iranian users at the beginning of the pandemic. The online search queries and the key terms searched by Iranian users varied during the COVID-19 pandemic. This study provides evidence in favor of the adoption of GT as an epidemiological surveillance tool but, it is necessary to consider that mass media and other confounders can significantly influence RSVs.

## Introduction

In December 2019, a novel coronavirus spread worldwide, causing the newly emerging coronavirus disease 2019 or COVID-19. This virus was first reported in Wuhan, Hubei Province, China [1]. COVID-19 is a controllable acute disease that can result in death in some cases. Based on the World Health Organization (WHO) reports, this virus has spread to all countries in the world. Over one hundred seventy-two million cases were reported by 3 June 2021, the majority of which belonged to the US and India. The disease rapidly grew in other countries [2, 3]. WHO reports that COVID-19 has a critical status and high statistics in Iran [2, 4]. The use of disease monitoring data during epidemics can significantly contribute to managing the situation [5]. Thus, all state-run and private institutions and organizations are making wide-ranging efforts to control the disease [6, 7].

The lack of behavior monitoring systems for patients and society during an outbreak is one of the main challenges in dealing with the outbreak of newly emerging diseases [8], such as COVID-19 [9]. In recent decades, extensive access to the Internet has made online social media and digital technologies a major source of information about the society, especially during epidemics [10, 11] when researchers and policymakers can use the collected Internet data. As a valuable auxiliary tool besides monitoring systems, these data help predict the outbreak of emerging diseases [12].

Every day, millions of people worldwide use online search engines (e.g., Google) to find health-related data and voluntarily share their health status and personal health-related behaviors. Thus, the data of tracking the behaviors of online health information searchers can be potentially applied in public health research and monitoring [12]. Google Trends (GT) is an important free tool for online search behavior analysis, which provides access to Internet search patterns in Google and offers deep insight into the online behavior of populations under study. In GT, users can specify their key terms, and GT shows the volume of these terms. Variations in the diagram reflect changes in the information search request by users or the key term’s usage over time [13–15]. Thus, as a timely, powerful, and sensitive monitoring system, GT can show clear indications about disease outbreaks. It is one of the best options for epidemics and outbreak monitoring and disease behavior trend analysis, in communities with a high Internet penetration factor [16, 17]. Based on a report published in January 2020, there are 58.42 million Internet users in Iran, which makes an Internet penetration factor of 70% [18–20]. Google is the most popular search engine among Iranian users, based on Alexa ranking [20]. Therefore, GT data analysis results can be used in national, regional, and provincial planning and policymaking to predict and fulfill the information needs in society [19–23].

In recent years, many studies have been conducted on online behavior analysis to predict the outbreak of diseases, especially viral diseases. The use of online behavior analysis tools has expanded in healthcare research. GT has been utilized as a time monitoring system to track many infectious diseases such as Lyme disease [19], tuberculosis [20], dengue [21], urinary tract infections [23], influenza [16, 23–27], AIDS [28, 29], and scarlet fever [30]. Identifying users’ online search behavior during outbreaks can significantly contribute to the management of outbreaks, providing easy access to information and predicting users’ information needs. Consequently, since the outset of the COVID-19 pandemic, numerous studies have examined users’ online COVID-19-related search behavior in many countries, such as the United States (US), India, Philippines, and Spain. The results of these studies have shown that GT search data can predict the trend of COVID-19 Outbreaks in different time lags [10, 31–34]. However, some studies highlighted the limitations and significant variations of GT for defining the epidemiology of diseases due to the influence of the mass media on web searches and the unpredictable fluctuations of the RSV [35, 36]. Moreover, a few studies found that the correlations between RSVs and COVID-19 cases have significant variations [36], and COVID-19–related GT data are more related to media coverage than epidemic data [37].

Thus, the present study aimed to evaluate Iranian users’ online search behavior during the COVID-19 pandemic and examine the relationship between users’ Internet search behavior for the COVID-19 pandemic and the epidemiological data of disease outbreaks.

## Methods

### Research design

This longitudinal study was conducted in 2021. The GT data of Iranian users were collected from 16 February 2020 to 2 January 2021 using the most frequently used search terms.

### GT data

The methodology of this study was designed based on the checklist proposed by Nuti et al. for documenting the use of GT. This checklist had been developed based on a systematic review of GT usage in healthcare to promote the quality and reliability of these studies [14]. First, the following key terms were searched on the GT website (https://trends.google.com/): (corona [Persian], Covid [Persian], COVID-19, corona, and coronavirus). By setting the country and the time range in exploring the section of the GT website, these key terms were extracted from the most related topics and queries in Iran in the study period. The search category was set to all categories. These terms were the most frequently used COVID-19-related terms among Iranian users. Then, the country (Iran) and period (from 16 February, when the first COVID-19 case was recorded in Iran, to 2 January 2021) were selected. The data of the terms’ relative popularity and the suggested topics in GT are based on the RSV per week. The RSV number shows the ratio of the popularity of a term or a topic relative to the peak popularity during a specific time in a selected region. RSV is reported on a 0–100 scale, where 100 means the term or topic has the highest level of popularity among the users, and 25 indicates that the search term or topic is 25% as popular as its peak popularity during the specified time in the specific region.

The data collection method complied with the terms and conditions of the Google Trends website.

### Epidemiological data

The number of new cases of COVID-19 in Iran from 16 February 2020 to 2 January 2021was retrieved from the website of the Iranian Ministry of Health and Medical Education (https://behdasht.gov.ir/) and cross-checked with the World Health Organization COVID-19 dashboard (https://covid19.who.int/region/emro/country/ir).

### Data analysis

The data were analyzed by using descriptive and analytical statistics. Initially, descriptive statistics were used to report the statistics of COVID-19 patients and the indices reported in GT. Since RSVs values have a high dependence on the day they were gathered, it is recommended that researchers should collect queries’ data for several consecutive days and deal with their RSVs averages instead of daily RSVs [36]. Thus, based on the longitudinal nature of the data, a marginal model was adopted to model the effect of the RSV of the five most frequently used COVID-19-related terms on the dependent variable (the number of new cases COVID-19). The generalized estimating equation (GEE) Negative binomial probability distribution, log link function, and exchangeable covariance matrix structure were utilized in this modeling for estimating parameters.

In this study, there was a high correlation between independent variables (RSV of search terms), and in regression models, it is assumed that the variables are independent. Therefore, before multivariate modeling, exploratory factor analysis was performed on the five independent variables to prevent the phenomenon of multiple collinearities and reduce it later (RSV of search terms). Data distribution was evaluated from different perspectives. The multivariate normality was evaluated using Mardia’s test, showing that the assumption was valid (c.r. = 1.723). Furthermore, the maximum value of skewness and kurtosis for the five variables were 1.940 and 4.305, respectively, and acceptable. Thus, the normality violation was not serious. The Mahalanobis distance was used to evaluate the outliner data, and its maximum value was 16.045. For df = 4, the Mahalanobis distance was lower than the critical value (18.47). So, there was no outliner data. Pearson correlation matrix for the five variables was sued to assess the linearity. The results suggested a robust linear correlation among all variables (S1 Appendix). Two factors were identified that correlate. Finally, the mentioned independent variables were determined in the form of factor score one and factor score two and entered the multivariate modeling stage. The scores of factor 1 and factor 2 were divided into quintiles, with the first quintile showing the lowest score of the factor and the fifth quintile indicating the highest score of that factor.

In the marginal model used, the dependent variable was patients with COVID-19. Independent variables are as follow: the first-factor quintile score (related to the higher RSV of the words Corona [Persian] and corona), the second-factor quintile score (related to the higher RSV of the words COVID [Persian] Corona Virus, and COVID-19), and time. Finally, in the final modeling, the main effect of the first factor quintile scores, the main effect of the second factor quintile scores, the main time effect, the interaction between the first factor quintile scores, and the time of the search, and the interaction between the second factor quintile scores and the search time, the number of cases of COVID-19 in Iran was investigated. This study was approved by the Lorestan University of Medical Sciences ethical committee (Ref no: IR.LUMS.REC.1399.011).

## Results

### Describing the study data

The results of the most important topics, queries, and sub-regions of the most frequent terms related to COVID-19 are presented in S2 Appendix.

**Fig 1:**
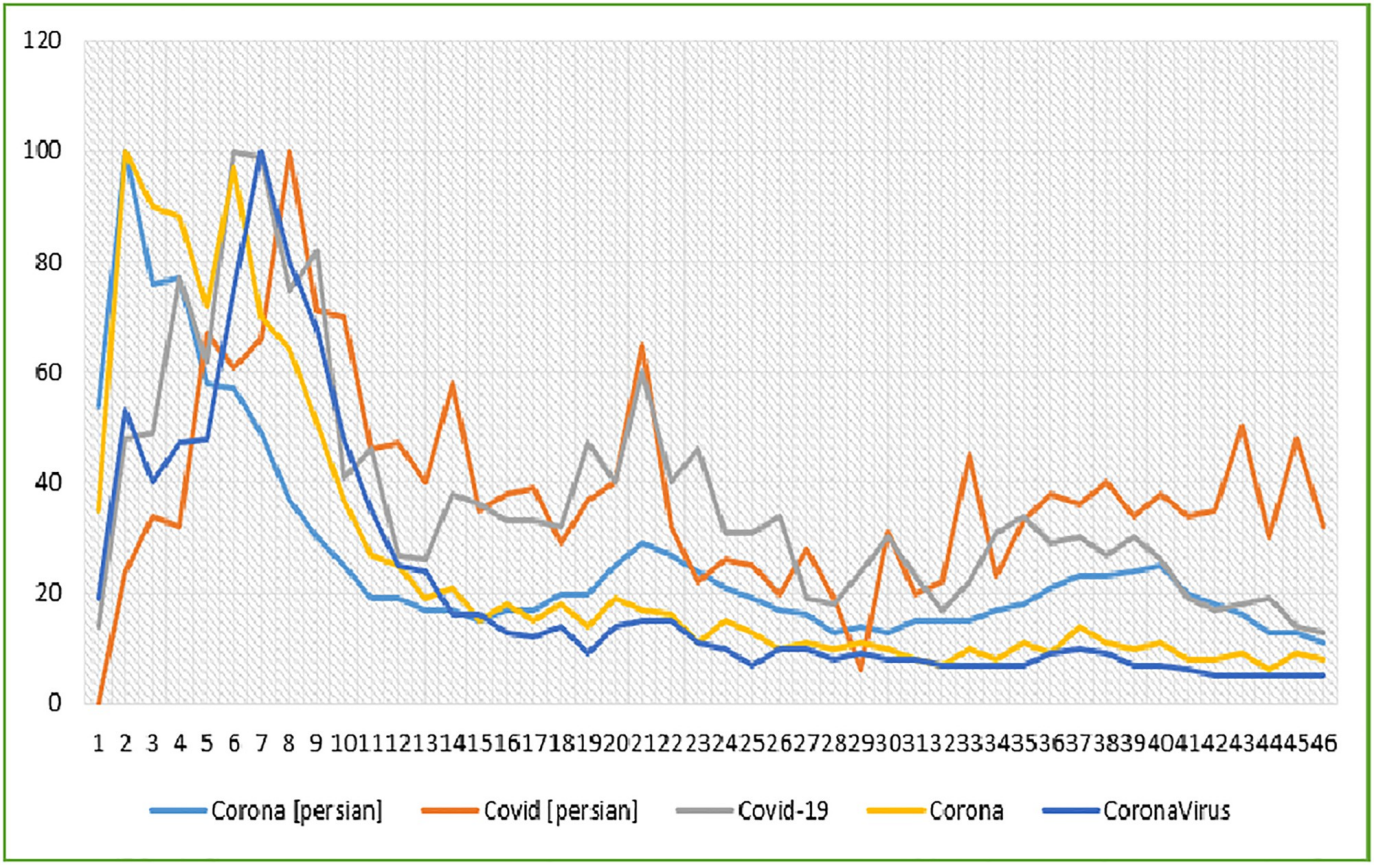
Search interest for COVID-19 related terms in weekly periods from 16 Feb 2020 to 2 Jan 2021.

Fig 1 displays the search interest of COVID-19-related terms in weekly periods from 16 February 2020 to 2 January 2021. The request for Internet search or the key terms searched by Iranian users varied during the COVID-19 pandemic.

**Fig 2:**
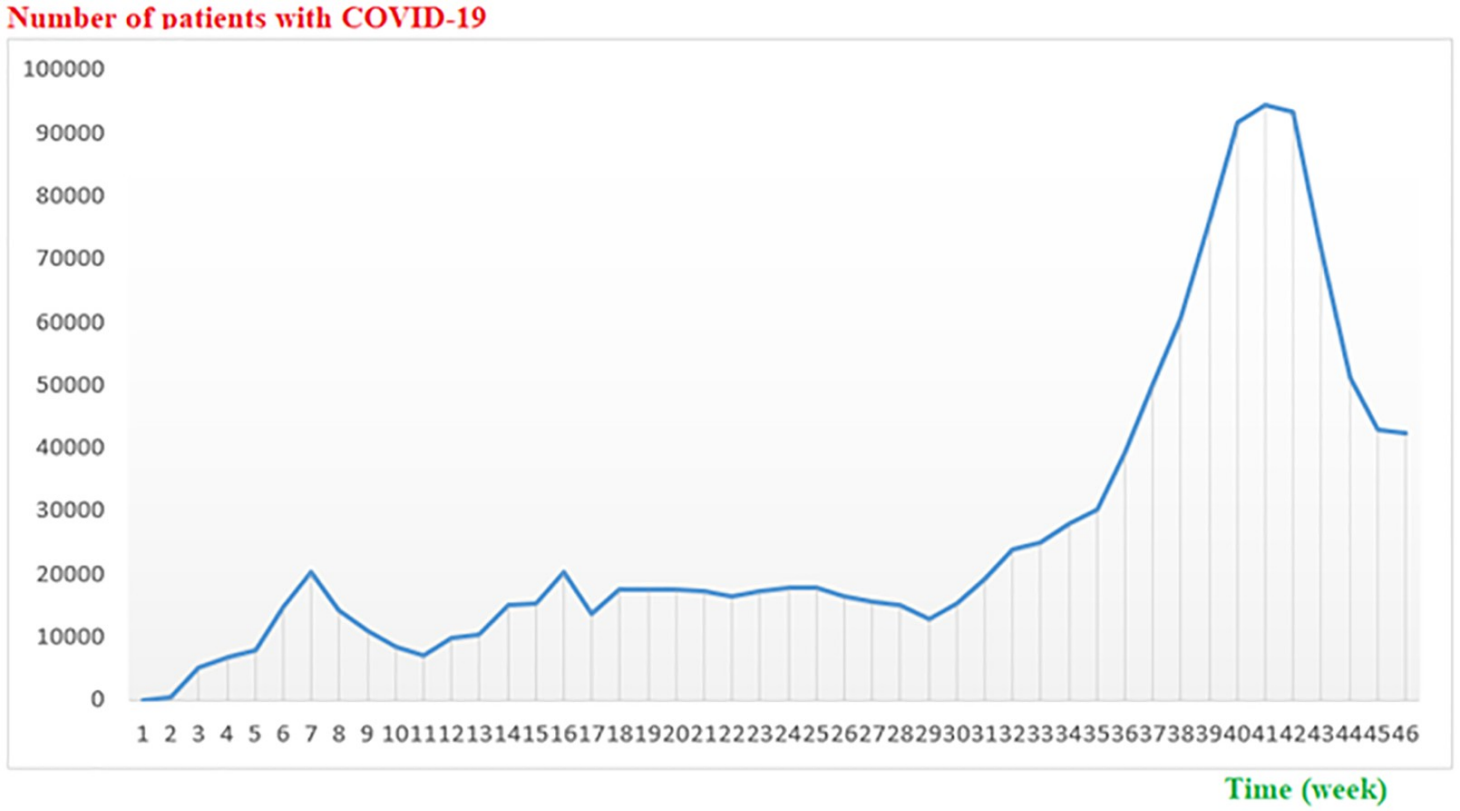
Weekly new cases of COVID-19 (16 Feb 2020 to 2 Jan 2021) in Iran.

Based on the findings of the weekly relative search volume (RSV) score, all the COVID-19-related search terms in Iran gained their highest popularity value (RSV = 100) in the first eight weeks of the pandemic, and then this value assumed a decreasing trend over time. The number of weekly new cases of COVID-19, from 16 February 2020 to 2 January 2021, in Iran, based on official statistics, has an increasing trend (Fig 2).

### Exploratory factor analysis on independent variables

The results of Bartlett’s test of sphericity and the Kaiser-Meyer-Olkin (KMO) index resulting from EFA, suggesting the adequacy of sampling (KMO = 0.726, X^2^ = 252.346, df = 10). Finally, two factors were extracted that explained 86.4% of the variance of the dependent variable (70.4% by factor 1 and 16.0% by factor 2). The pattern matrix indicated that factor 1 was related to corona (Persian) search (factor loading: 1.036) and corona search (factor loading: 0.845). Moreover, factor 2 was closely related to Covid (Persian) search (factor loading: 0.839), coronavirus search (factor loading: 0.707), and COVID-19 search (factor loading: 0.686). The first quintile of factor score 1 denoted the lowest search score for corona [Persian] and corona, while the fifth quintile of factor score 2 denoted the highest search score for Covid [Persian], coronavirus, and COVID-19.

### Marginal modeling

Table 1 presents the median and inter-quartile range (IQR) of factor score 1 and factor score 2 based on the quintiles of factors 1 and factor 2 in different time lags within the range of [7, 21] days.

**Table 1:** Mean and IQR of factor score 1 and factor score 2 based on quintiles.

Factors		Epidemiological data (Cases of Covid-19) (time)					
		1 week		2 weeks		3 weeks	
		Median	IQR	Median	IQR	Median	IQR
Factor 1	quintile 1	28133.00	18848.00	30237.00	17858.00	39215.00	14378.00
	quintile 2	20477.00	23493.00	19103.00	35043.00	24043.00	32438.00
	quintile 3	17637.50	34638.00	17653.00	44100.00	17418.00	60553.00
	quintile 4	17233.00	7588.00	17233.00	2811.00	17233.00	2511.00
	quintile 5	8460.00	7420.00	8460.00	7166.00	9772.00	6405.00
Factor 2	quintile 1	42891.00	67740.00	42511.00	46799.00	42891.00	23121.00
	quintile 2	42964.00	34876.00	42891.00	44756.00	42511.00	57172.00
	quintile 3	17926.50	10900.00	17926.00	13859.00	17589.00	23493.00
	quintile 4	16489.00	2500.00	17233.00	2168.00	17597.00	1189.00
	quintile 5	10325.00	5826.00	10839.00	5026.00	10839.00	5357.00

**Fig 3:**
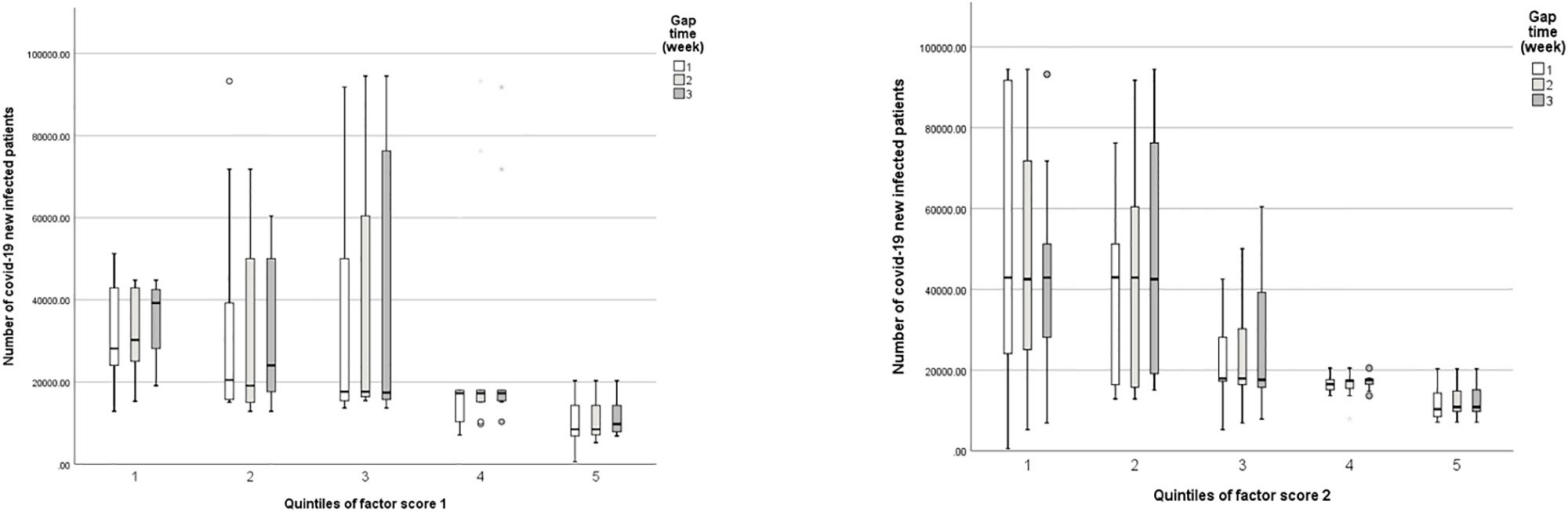
Box plot of quintiles in factor score 1 and factor score 2 based on time.

Also, Fig 3 depicts the box plot of the quintiles of factor score 1 and factor score 2 per time.

The results of the model are shown in Table 2. According to the marginal model, the interaction of factor scores “1” quintiles (search score for corona [Persian] and corona (and time on the number of COVID-19 patients was low significant (X^2^ = 2.091, df = 4, P = 0.719). Furthermore, the main effect of the quintiles of factor score “1” on the number of COVID-19 patients was low significant but considerable (X^2^ = 7.419, df = 4, P = 0.115). Although, the factor scores “2” quintiles (search score for Covid [Persian], coronavirus, and COVID-19) and time interaction on the number of COVID-19 patients was of high significance (X^2^ = 10.094, df = 4, P = 0.039).

**Table 2:** The effect of the marginal model results in the independent and dependent variables.

Source			
	X^2^	df	P
Lag Time (week)	2.166	1	0.141
Factor 1. quintile	7.419	4	0.115
Factor 2. quintile	26.794	4	<0.001
Factor 1. Quintile * Time (week)	2.091	4	0.719
Factor 2. quintile * Time (week)	10.094	4	0.039

This rate ratio (RR) of COVID-19 infection was 16.4% higher in the second quintile of factor score "1" compared to the first quintile of factor score "1", and it was low significant statistically (RR = 1.164, 95% CI = 0.684–1.980, p = 0.576). Table 3 presents the other information about parameter estimates.

**Table 3:** Parameter estimates of model.

Parameter	Parameter estimate									
	B	S. E	95% CI interval		Hypothesis Test			RR	95% CI interval for RR	
			Lower	Upper	X^2^	Df	p		Lower	Upper
[factor1.quintile = 5] * Time (week)	.095	.0927	-.086	.277	1.059	1	.303	1.100	.917	1.319
[factor1.quintile = 4] * Time (week)	.039	.0653	-.089	.167	.349	1	.554	1.039	.914	1.181
[factor1.quintile = 3] * Time (week)	.006	.0539	-.100	.112	.012	1	.913	1.006	.905	1.118
[factor1.quintile = 2] * Time (week)	-.042	.0745	-.188	.104	.320	1	.572	.959	.829	1.109
[factor1.quintile = 1] * Time (week)	0^a^	.	.	.	.	.	.	1	.	.
[factor2.quintile = 5] * Time (week)	-.008	.1090	-.221	.206	.005	1	.945	.993	.802	1.229
[factor2.quintile = 4] * Time (week)	.119	.0708	-.019	.258	2.847	1	.092	1.127	.981	1.295
[factor2.quintile = 3] * Time (week)	.151	.0695	.014	.287	4.697	1	.030	1.163	1.015	1.332
[factor2.quintile = 2] * Time (week)	.176	.0663	.046	.306	7.063	1	.008	1.193	1.047	1.358
[factor2.quintile = 1] * Time (week)	0^a^	.0514	.	.	.	.	.	1	.	.

The marginal model also revealed that the factor score “2” quintiles and time interaction on the number of COVID-19 patients were highly significant (X^2^ = 10.094, df = 4, P = 0.039). Thus, Table 4 shows the relative rate of COVID-19 incidence in one-, two-, and three-week time lags in factor score 2.

**Table 4:** COVID-19 incidence rate ratio.

Type of quintile	The formula for relative rate of COVID-19 infection per time interval	The rate ratio of COVID-19 infection		
		t = 1	t = 2	t = 3
Quintile 2, factor 2 (compared to quintile 1)	Exp^(-0.446+0.176^*^t)^	1.087	0.911	0.764
Quintile 3, factor 2 (compared to quintile 1)	Exp^(-0.949+0.151^*^t)^	0.450	0.523	0.608
Quintile 4, factor 2 (compared to quintile 1)	Exp^(-1.302+0.119^*^t)^	0.307	0.345	0.389
Quintile 5, factor 2 (compared to quintile 1)	Exp^(-0.861–0.008^*^t)^	0.419	0.416	0.413

## Discussion

Since GT data are real and produced at the right time, they can help accurately monitor various diseases in different regions. Moreover, studies have demonstrated that specific word searches in the Google search engine may predict new suspected COVID-19 case numbers. Whereas, GT has the many advantages of being measured in near real-time, available across a wide range of geographical locations, and not prone to desirability biases, some notable limitations are presented on COVID-19. These limitations include measuring search patterns and not the actual user behaviors, providing data in the form of RSV instead of absolute search volumes and its granularity [38–40]. Therefore, caution should be exercised when interpreting the results of GT.

The present study examined the COVID-19 related online search behavior of Iranian users using GT and investigated its relationship with COVID-19 incidence during the pandemic. Based on the results, the weekly RSV of all the COVID-19-related search terms reached their highest popularity value during the first eight weeks of the pandemic, and then this value assumed a decreasing trend. Similarly, after the COVID-19 outbreak and reporting the first cases in different countries, Google users’ search of this disease rapidly increased [41]. A study observed a sharp increase in the GT from 10th March to 10th April 2020 in eight major countries (United States, Spain, Italy, France, United Kingdom, China, Iran, and India) [42]. Also, GT for wash hand face mask had rapidly increased in COVID-19 outbreak among 21 countries [33].

Moreover, GT data had the swift growth of the second wave of interest in COVID-19 since 21 February 2020. This rising interest trend was observed worldwide and in the presented countries, where a rapid increase in cases of laboratory-confirmed COVID-19 has been reported since 21 February 2020. Several studies on users’ online search behavior for COVID-19 showed that GT data could predict the trend of the outbreak [9, 31–34, 41, 43–48]. The GT service works as a data reverse engineering tool and helps collect data on public interests [37]. Thus, the governments can utilize the GT to improve their health service management using timely information. It is recommended that healthcare policymakers and authorities realize the importance of GT-based analyses in determining the public’s information needs and planning to provide valid data accordingly [31]. However, the decreasing trend of RSV among Iranian users after the first peek of the COVID-19 pandemic can be due to the users’ gradual tendency towards online social media to seek their required health information. Many people tend to use these media to allow easy, dynamic, and continuous access to information by social media [49].

A study conducted to predict the COVID-19 outbreak using GT in India recommended that Google search data be used as a complementary tool for COVID-19 control and management programs [34]. Our results showed that RSV of factor score one term related to corona [Persian] and corona have a low significant relationship with COVID-19 epidemiological data. Similarly, no correlation was observed between the RSV regarding COVID-19 related terms in the USA and the total number of infected patients in other countries (except China) [50]. Also, low significance correlations were found between daily RSVs related to loss of smell with the increases of daily confirmed cases of COVID-19 1st of January and 1st of February 2020 [51]. Although, RSV of factor 2 terms was related to more specialized search terms (Covid [Persian], COVID-19, and coronavirus), and correlated with the total weekly number of COVID-19 cases in one-, two-, and three-week time lags. Similarly, according to the studies conducted in different countries, Internet search data related to the COVID-19 pandemic had a high significance positive relationship with the number of COVID-19 cases in different time lags [31, 33, 34, 41, 44–48, 51, 52]. The growth trend of COVID-19 could be predicted based on GT data. These results are meaningful for different stakeholders and can be used in planning and policymaking to predict and accomplish the information needs in society.

Also, according to a recent study [50], the real-world confirmed cases with COVID-19 were strongly correlated with search terms of COVID-19, coronavirus, coronavirus, and SARS-CoV-2. In addition, even though outbreaks have occurred at different times in different countries, the relationships between the search terms and identified COVID-19 cases remain similar across countries [48]. Based on the results, the Iranian users have different behavior in search terms against other countries [48, 51, 53]. However, no information is available on the users’ socioeconomic status and education level, the users searching specialized key terms related to factor 2 have a higher education level. Although various factors should be examined to identify and predict the COVID-19 outbreak, like any other pandemic, GT can be used as an accessible, easy, dynamic, and real method for predicting the COVID-19 outbreak [54]. There were limitations in terms of data collection and the accuracy assurance of epidemiological data related to the COVID-19 in Iran. However, this source (GT score) can still be used as an auxiliary online source of information in the first days and weeks of outbreaks for predicting outbreak trends. Moreover, this valuable complementary source can also be used for population tracking of pandemics and help improve public health response, particularly during the early stages of a pandemic.

### Limitations of the study

The study does not take into account the potential spurious correlations due to the influence of the mass and social media on web searches and information-seeking behavior, which might influence the results of the study to an extent. Future studies might consider using techniques for cleaning data from the influence of the mass media.

## Supporting information

S1 Appendix Pearson correlation matrix of study variables. (DOCX)

S2 Appendix Topics, queries, and sub-regions of important search terms related to COVID-19. (DOCX)
