# Supplementary material for: Assessing the online search behavior for COVID-19 outbreak: Evidence from Iran
Source: PLoS One. 2022 Jul 26;17(7):e0267818. doi: 10.1371/journal.pone.0267818 (PMC9321440; doi:10.1371/journal.pone.0267818)
Supplement: S1 Appendix — (DOCX) [file pone.0267818.s001.docx]

Appendix 1. Pearson correlation matrix of study variables

|  | | Corona | covid | COVID19 | Corona (Eng.) | coronavirus |
| --- | --- | --- | --- | --- | --- | --- |
| Corona | PC | 1 | 0.122 | 0.580 | 0.915 | 0.542 |
|  | Sig. (2-tailed) |  | 0.153 | 0.000 | 0.000 | 0.000 |
| covid | PC | 0.122 | 1 | 0.609 | 0.383 | 0.635 |
|  | Sig. (2-tailed) | 0.153 |  | 0.000 | 0.000 | 0.000 |
| COVID19 | PC | 0.580 | 0.609 | 1 | 0.768 | 0.874 |
|  | Sig. (2-tailed) | 0.000 | 0.000 |  | 0.000 | 0.000 |
| Corona (Eng.) | PC | 0.915 | 0.383 | 0.768 | 1 | 0.850 |
|  | Sig. (2-tailed) | 0.000 | 0.000 | 0.000 |  | 0.000 |
| coronavirus | PC | 0.642 | 0.635 | 0.874 | 0.850 | 1 |
|  | Sig. (2-tailed) | 0.000 | 0.000 | 0.000 | 0.000 |  |

PC: Pearson Correlation
