# Supplementary material for: Assessing the online search behavior for COVID-19 outbreak: Evidence from Iran
Source: PLoS One. 2022 Jul 26;17(7):e0267818. doi: 10.1371/journal.pone.0267818 (PMC9321440; doi:10.1371/journal.pone.0267818)
Supplement: S2 Appendix — (DOCX) [file pone.0267818.s002.docx]

Appendix 2. Topics, queries, and sub-regions of important search terms related to COVID-19

| **Search terms corona [Persian]** | | | |
| --- | --- | --- | --- |
| **Related topics** | **Related queries** | **Subregion** | RSV |
| Statistics – Discipline | [symptoms of corona] | Chahrmahal and Bakhtiari | 100 |
| Vaccine – Topic | [corona Iran] | KohgiluyeshBoyar Ahmad | 97 |
| Signs and symptoms- Topic | [corona vaccine] | Lorestan | 95 |
| Coronavirus-Virus | [corona statistics] | Kerman | 94 |
| Virus-Infectious agent | [corona today] | Fars | 88 |
| **Search terms covid [Persian]** | | | |
| **Related topics** | **Related queries** | **Subregion** | RSV |
| Coronavirus disease 2019-Disease | [COVID-19] | South Khorasan | 100 |
| Coronavirus-Virus | [COVID19] | Qazvin | 68 |
| Virus-infectious agent | [hey, COVID-19] | North Khorasan | 65 |
| Disease-Topic | [songs about COVID-19] | Charmahal Bakhtiari | 54 |
| Experiment-Topic | [hey, COVID19] | Markazi | 52 |
| **Search terms [Covid 19]** | | | |
| **Related topics** | **Related queries** | **Subregion** | RSV |
| Coronavirus disease 2019-Disease | Corona | Tehran | 100 |
| Coronavirus-Virus | covid 19 Vaccine | Kurdistan | 76 |
| Vaccine-Topic | Coronavirus | Mazandaran | 75 |
| Statistics-Discipline | Covid 19 map | South Khorasan | 72 |
| Covid-19vaccine-pharmaceutical | Covid 19 statistics | Zanjan | 68 |
| **Search terms [Corona]** | | | |
| **Related topics** | **Related queries** | **Subregion** | RSV |
| Virus-Infectious agent | Corona virus | Tehran | 100 |
| Statistics-Discipline | Statistics corona | Qazvin | 68 |
| Death-Topic | Coronavirus | Alborz | 66 |
| Worldmeters-Website | corona world | Lorestan | 62 |
| Coronavirus disease 2019-Disease | corona iran | Mazandaran | 61 |
| **Search terms [Coronavirus]** | | | |
| **Related topics** | **Related queries** | **Subregion** | RSV |
| Statistics-Discipline | corona [Persian] | Tehran | 100 |
| Worldmeters-Website | Statistics coronavirus | Qazvin | 77 |
| Virus-Infectious agent | Coronavirus world | West Azerbaijan | 72 |
| Vaccine-Topic | Coronavirus update | Hamadan | 69 |
| Coronavirus disease 2019-Disease | Coronavirus worldmeter | Alborz | 67 |
